# Aging and respiratory viral infection: from acute morbidity to chronic sequelae

**DOI:** 10.1186/s13578-021-00624-2

**Published:** 2021-06-22

**Authors:** Yue Wu, Nick P. Goplen, Jie Sun

**Affiliations:** 1 Department of Immunology, Mayo Clinic, Rochester, MN 55905 USA; 2 Division of Pulmonary and Critical Medicine, Department of Medicine, Mayo Clinic, Rochester, MN 55905 USA; 3 The Robert and Arlene Kogod Center on Aging, Mayo Clinic, Rochester, MN 55905 USA; 4 Department of Physiology and Biomedical Engineering, Mayo Clinic, Rochester, MN 55905 USA

**Keywords:** Aging, Respiratory viral infections, Influenza virus infection, COVID-19, Immune response

## Abstract

The altered immune response in aged hosts play a vital role in contributing to their increased morbidity and mortality during respiratory virus infections. The aged hosts display impaired antiviral immune response as well as increased risk for long-term pulmonary sequelae post virus clearance. However, the underlying cellular and molecular mechanisms driving these alterations of the immune compartment have not been fully elucidated. During the era of COVID-19 pandemic, a better understanding of such aspects is urgently needed to provide insight that will benefit the geriatric patient care in prevention as well as treatment. Here, we review the current knowledge about the unique immune characteristics of aged hosts during homeostasis and respiratory virus infections.

## Introduction

Aging is a major factor that contributes to the increased morbidity and mortality during respiratory virus infections including influenza virus and severe acute respiratory syndrome coronavirus 2 (SARS-CoV-2) infection [1–3]. By the year 2050, the global aged population (65 years and over) will double to ~ 1.5 billion. [4] Such outlook necessitates the development of strategies to improve the quality of life for the elderly. Although the cellular and molecular mechanisms that lead to such phenomena have yet to be fully elucidated, accumulating evidence suggests that the immune system plays an indispensable role in the unique pathophysiological process of respiratory virus infection in the aged hosts. In this review, we provide a brief overview of the current knowledge of the altered immune response in aged hosts during homeostasis and respiratory virus infection. Moreover, we discuss the role of the immune response in long-term lung sequelae following viral pneumonia. To gain better insight, we will first use the influenza virus infection model due to the depth of prior research, followed by a discussion about current knowledge on the immune response towards SARS-CoV2 emphasizing the aging element.

## The aging immune system

The life-long maintenance of most immune cell populations is supplied by the Hematopoietic Stem Cells (HSC) and their progenitors that reside in the bone marrow. Despite the age-associated increase in the frequency of HSCs, aging impairs the capacity of HSCs to self-renew and imposes a myeloid bias limiting their multipotency relative to young counterparts [5, 6]. The HSCs in aged hosts face declining self-renewal ability, which is associated with increased DNA damage [7]. Such defects in HSCs could be induced by replicative stress, as implicated in serial transplantation models [7], as well as oxidative stress, represented by increased ROS level in the aged HSCs [8]. Heterochronic transplantation of bone marrow cells from aged donors to young hosts or vice versa suggests a role for environmental factors in the aging phenotype of HSCs [9–12].

**Fig. 1:**
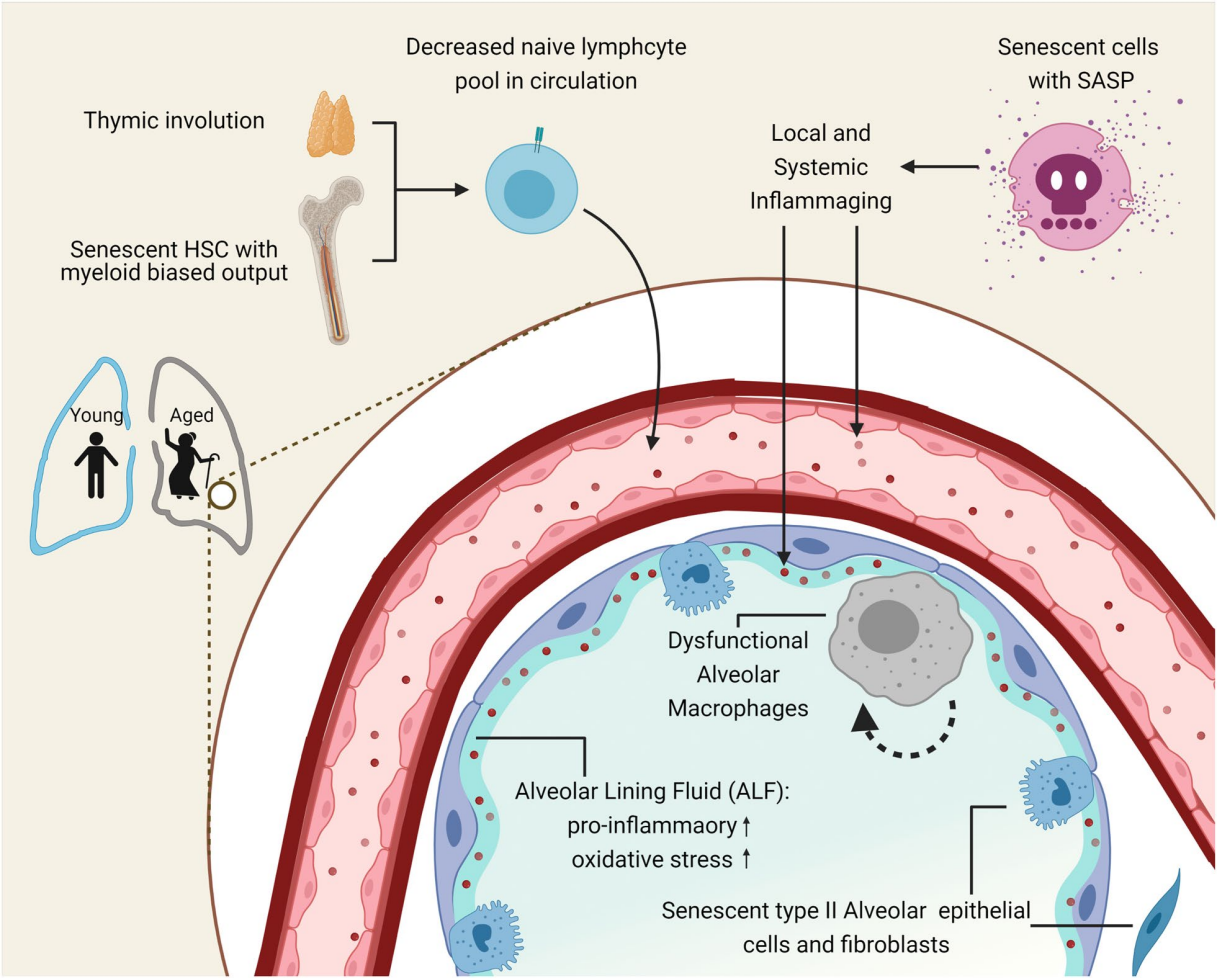
Immune alteration during physiological aging. Physiological aging is associated with a series of immune-related modifications that could contribute to age-associated diseases like respiratory virus infections. Inflammaging describes a progressing low-level inflammation associated with age. A feed-forward circuit is formed between senescent cells and inflammaging. In the lung, dysfunctional surfactant turnover by type II airway epithelial cells (AT II) and alveolar macrophages (AMs) could also lead to persistence of oxidative and pro-inflammatory molecules in the alveolar lining fluid. Moreover, an age-associated decline in the naïve lymphocyte pool, attributed to myeloid bias of HSC cells and thymic involution, could abrogate successful defense against respiratory viruses

One of the main criteria for a successful defense system against a new pathogen is a naïve T cell pool with high diversity and abundant cell quantity. However, the proportion of naïve T cells decreases significantly in both circulation and lymphoid tissue with aging (Fig. 1). This is partially contributed by thymic involution, which causes a steep decline of thymic output with aging [13, 14]. It has been reported that in people over age 40, the thymus had a tenfold decline in output, and the naïve T cell maintenance may be more dependent on homeostatic clonal expansion [15]. Moreover, there is a site-specific bias in such clonal expansion, which could lead to decreased TCR diversity [15]. Taken together, the naïve T cell number and diversity decrease with age, which could put the aged hosts at risk of impaired T cell immunity against new pathogens such as SARS-CoV-2.

Inflammaging, presenting as chronic low-level inflammation in aged individuals, is one of the main contributors to age-associated diseases [16] (Fig. 1). Cellular senescence describes a process characterized by permanent cell cycle arrest and gain of senescence-associated secretory phenotype (SASP), a pro-inflammatory secretome [17]. It has been observed that in the alveolar lining fluid (ALF) of the aged hosts, there was an increased presence of innate cytokines (IL-6, IL1-β), the components of the classical complement pathway, and indicators of oxidative stress (such as Myeloperoxidase and oxidized lipids) [18]. Such phenotype could also be attributed to age-associated dysfunction in phagocytosis ability of alveolar macrophages (AMs) [19], which are important for surfactant turn-over and clearance of the pro-inflammatory cellular and molecular components. The oxidative and pro-inflammatory environment in the aged hosts could alter the immune cell behavior needed for the relative immune-suppressive lung environment during homeostasis. Such environmental alterations may influence the length and scale of the immune response as well as the tissue damage and repair processes during respiratory viral infections.

## Aging and innate immunity against respiratory viral infections

**Fig. 2:**
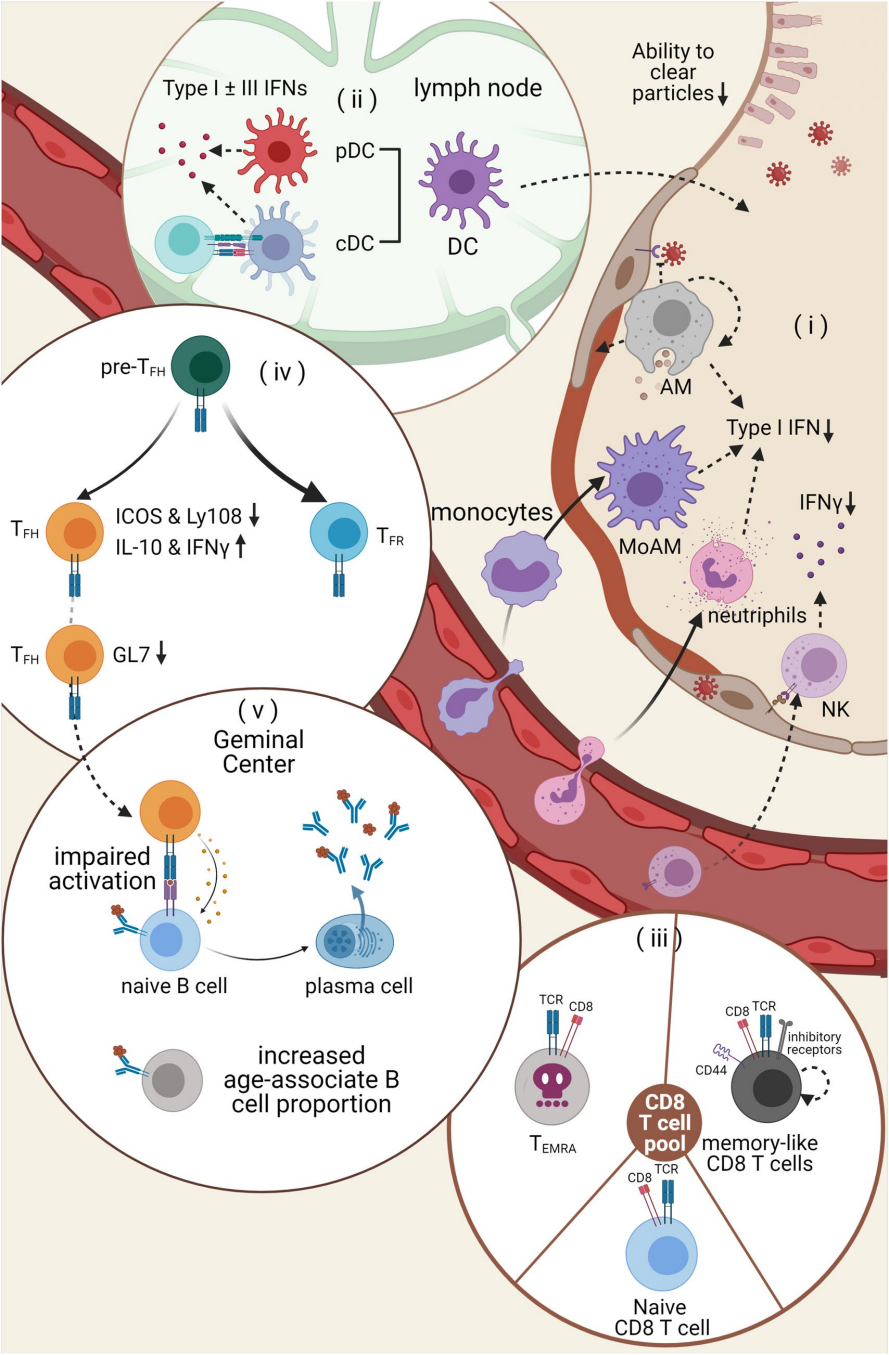
Aging is associate with dysfunctional anti-viral immunity. **a**,** b** Impaired innate immunity against respiratory virus infection. **a** The aged hosts have decreased capacity to clear foreign bodies including the virus particles. The production of key anti-viral cytokines like Type I IFN is also abrogated with progressing age. Besides the cytokine production, reduced AM self-renewal also hinders anti-viral response because of AMs’ ability to prevent the ATI cells from the virus infection and to phagocytose virus and virus-infected cells. Additionally, the dysfunction of NK cell infiltration and function also contributes to the defect. **b** DCs also contribute to the malfunctional anti-viral response in the elderly. Aged lung has increased PGD2 level, which abrogates cDC’ migration to the lymph node, where they prime T cell responses. Moreover, both cDCs and pDCs have reduced production of Type I and /or type III IFNs upon viral stimulation. **c**–**e** Aging compromises the adaptive immunity against virus infection. **c** Aging is associated with decline in the total size of naïve T cell pool, accompanied by accumulation of memory-like CD8 T cell and terminally differentiated CD8^+^ effector cell (T_EMRA_) population. Both the populations display reduced repertoire diversity, which limits the effective cellular response in the aged host towards new virus infection. **d** Dysfunctional CD4 T cell help contribute to the impaired humoral response in the aged host. Activated CD4 T cells have a fate choice biased towards T_FR_ instead of T_FH_. And they displayed reduce migration towards the Germinal Center. **e** Age-associated B cells accumulate with progressing age, accompanied by suboptimal humoral response following respiratory viral infection and/or vaccination

Aging is associated with increased susceptibility towards respiratory viral infections, which is likely attributed to the decreased vaccine efficacy in the elderly and/or the impaired first-line defense in the lung. The first line of immune defense is a broad spectrum of barriers including physical, chemical and cellular components (Fig. 2a). Capacity to clear foreign bodies from the airways declines with age because of the progressive deterioration of respiratory muscle strength, cough strength, and function of mucociliary cells that clear particles in the upper and lower airways [20]. The alveoli are the interface of inner and outer environments. The integrity of the air-blood barrier in alveoli as well as the appropriate composition of ALF is essential for first-line defense. Despite the lack of evidence suggesting an age-associated difference in the permeability of the airway barrier, it has been shown that aging exacerbated the increase of protein level in the bronchial alveolar lavage fluid (BAL) upon LPS stimulation [21], indicating that aging may affect the physiological responses of the alveoli to microbial invasion.

Induction of antiviral innate immune response, particularly the production of the anti-viral cytokines like type I and/or III interferons (IFNs), is impaired during aging despite the myeloid bias in hematopoiesis and increased baseline activation of innate immune cells (Fig. 2b). Plasmacytoid dendritic cells (pDCs), whose number decline with aging, are a major cellular source of type I IFNs following viral infection [22]. pDCs from aged hosts showed impaired type I IFN production after stimulation with influenza virus or TLR ligands, potentially due to the impaired phosphorylation of IRF7 [23]. Conventional dendritic cells (cDCs) are the major professional antigen-presenting cells for the activation of anti-viral T cells. In vitro, cDCs from the elderly displayed impaired production of type I and type III IFNs following the exposure to influenza virus [24, 25]. In vivo, aged lungs had increased levels of prostaglandin D2 (PGD2), causing decreased migration of cDCs to the draining lymph nodes, thereby compromising the development of the anti-viral immunity in the aged hosts [26]. The abrogated pDC and cDC function is believed to be at least partially responsible for the delayed induction and decreased magnitude of antiviral immune responses observed during aging.

In addition to DCs, prompt induction of antiviral immune response in monocytes and macrophages is crucial in limiting respiratory viral replication (Fig. 2a). It has been shown that monocytes from the elderly had a reduced level of TRAF-3 [27], a critical adaptor molecule downstream of the RIG-I signaling [28, 29]. As a result, monocytes from the elderly showed impaired type I IFN production upon RIG-I ligand stimulation [27]. Monocytes and monocytes-derived macrophages can contribute to anti-viral immunity at the potential cost of causing significant lung inflammation and injury during influenza virus infection [30]. In such context, aged mice displayed prolonged infiltration of monocytes and monocytes-derived macrophages following influenza virus infection, which may contribute to the increased disease development during aging [31]. Besides monocytes and monocytes-derived macrophages, aging drastically increases neutrophil infiltration to the lungs following influenza virus infection, potentially due to the increased production of neutrophil chemoattractants (CXCL1 and CXCL2) in aged lungs [32]. Importantly, the depletion of neutrophils starting on day 6 post-infection substantially improved the survival of aged mice without altering viral clearance [32], suggesting that excessive neutrophil responses are a key driver of the enhanced host diseases observed in aged hosts following influenza virus infection [30, 33].

Alveolar macrophages (AMs) are the major immune cells that reside in the lung during homeostasis and maintain the immune homeostasis of the lung during steady state [34]. AMs are a major cellular source of type I IFNs during certain respiratory viral infections [35]. Furthermore, AMs can phagocytose virus and virus-infected cells and protect type I Alveolar epithelial cells (ATI) from infection to maintain critical lung physiological function following respiratory viral infection [35–37] (Fig. 2a). Thus, AMs play an indispensable role in host defense against respiratory viral infection. Notably, AMs from aged mice had reduced proliferative capacity, potentially contributing to the reduced numbers of AMs observed in the aged lungs [19, 38]. Moreover, AMs from the aged hosts had altered phenotypes and impaired phagocytic ability compared to those from the young [19]. Importantly, the transfer of AMs from young mice into aged hosts partially ameliorated the enhanced disease development following influenza infection, suggesting that impaired AM abundance and function partially contribute to the enhanced susceptibility to severe influenza infection [19].

Another important anti-viral effector cell type is natural killer (NK) cells, which eliminate virus-infected cells by their cytotoxic function and/or the production of anti-viral cytokines including IFN-γ (Fig. 2a). The level of circulating NK cells decreases in the aged hosts during steady state, and these NK cells display a terminally differentiated phenotype when compared to NK cells from young adults [39, 40]. During influenza virus infection, reduced and delayed infiltration of NK cells, especially the activated NK cells, was observed in aged hosts [31]. Additionally, the activation of NK cells, indicated by the expression of CD69, is impaired [31, 41]. Collectively, aging profoundly impairs anti-viral innate immunity in the respiratory tract, which results in increased susceptibility to respiratory viral infection, decreased capacity to restrain virus and/or increased tissue injury. Future interventions aimed at improving the function of the aged innate immune system may be beneficial to prevent and/or treat severe respiratory viral infections in the elderly.

## Aging and adaptive immunity against viral infection

As discussed above, aging reduces the total size of the naïve T cell pool and the clonal diversity of T cells (Fig. 2c). This is particularly true for influenza-specific T cells as studies have demonstrated that influenza antigen-specific naïve precursors were downregulated up to tenfold during aging [42]. Furthermore, it has been reported that aging shifted the TCR-β usage of naïve T cells against a major influenza epitope, leading to the age-associated loss in the responsiveness in CD8 T cell expansion during infection [43]. Accompanied by the decreased naïve T cell pool, aging is associated with the drastically increased presence of memory-like naive T cells. For instance, the majority of naive CD8 T cells from uninfected aged mice express CD44, a marker for activated or memory T cells, and have features of antigen-experienced memory T cells [42]. These age-associated memory-like T cells have increased expression of inhibitory receptors (PD1, LAG3 and 2B4), inefficient clonal expansion and poor accumulation in the blood following viral infection [42]. Notably, the response of influenza-naive aged mice to influenza infection is mediated largely by those memory-like CD8 T cells that have reduced repertoire diversity [44], which may contribute to the dysregulated T cell responses observed in aged hosts. Similarly, the expansion of memory and terminally differentiated CD8^+^ effector cell (T_EMRA_) populations were observed during human aging [45]. Those T_EMRA_ cells were considered to possess a senescence-like phenotype, characterized by progressive loss of costimulatory signal receptor (CD27 and CD28), low telomerase activity, activated senescence-related pro-inflammatory pathways (i.e. MAPK pathways) and dysfunctional mitochondria [46]. Notably, the frequency of CD8 + T_EMRA_ cells in the circulation is inversely correlated with the antibody response following influenza vaccine, indicating potential detrimental effects of T_EMRA_ cells for de novo induction of immune responses following vaccination or viral infection in aged hosts [45].

The generation of robust CD8 T cell and B cell responses require CD4 T cell help in the context of influenza virus infection. To this end, the quantity and function of follicular helper T (Tfh) cells, a specialized T helper cell population that is required for the germinal center B cell responses and class-switched high-affinity antibody production, are impaired during aging (Fig. 2d). In animal studies, it has been shown that the activated influenza-specific CD4 T cells were able to upregulate Bcl6 and become “T_FH_-committed” cells in the aged hosts. However, these “pre-T_FH_ cells” failed to upregulate the expression of ICOS and SLAMF6, nor were they able to increase GL7 expression, which is required for migration into the germinal center, where T-B interactions take place. Instead, they displayed elevated production of IL-10 and IFN-γ, which potentially impaired their interaction with cognate B cells for high affinity antibody production [47]. Additionally, activated CD4 T cells from aged mice committed to the fate of T follicular regulatory (T_FR_) cells, which functionally oppose T_FH_. Consequently, influenza-specific IgM and IgG production were reduced during aging [47, 48] (Fig. 2e). In line with the impaired T_FH_ responses in aged animals following influenza infection and/or vaccine, elderly humans have reduced circulating Tfh-like (cT_FH_) cells in the blood and those cells have a per-cell decrease in functional ability to help B cells compared with cT_FH_ from young adults [49].

Like T cells, it has been shown that the absolute number of B cells decreased with age [50]. Interestingly, a unique mature B cell population termed age-associated B cells (ABCs) was found to accumulate with age (Fig. 2e). They were found to secrete antibody as well as cytokines (IFN-γ, IL-4, IL-6 and IL-10) preferentially upon TLR7 or TLR9 ligation rather than BCR activation [51, 52]. Upon in vitro stimulation of anti-CD40/IL-4, the production of IgG decreased with age in B cells, indicating that class-switch recombination (CSR) declines with age [50]. Together, the age-associated impairment in B and CD4 T cell function ultimately contributes to suboptimal humoral responses following respiratory viral infection and/or vaccination in the elderly.

## Age-associated prolonged recovery following respiratory viral infection

**Fig. 3:**
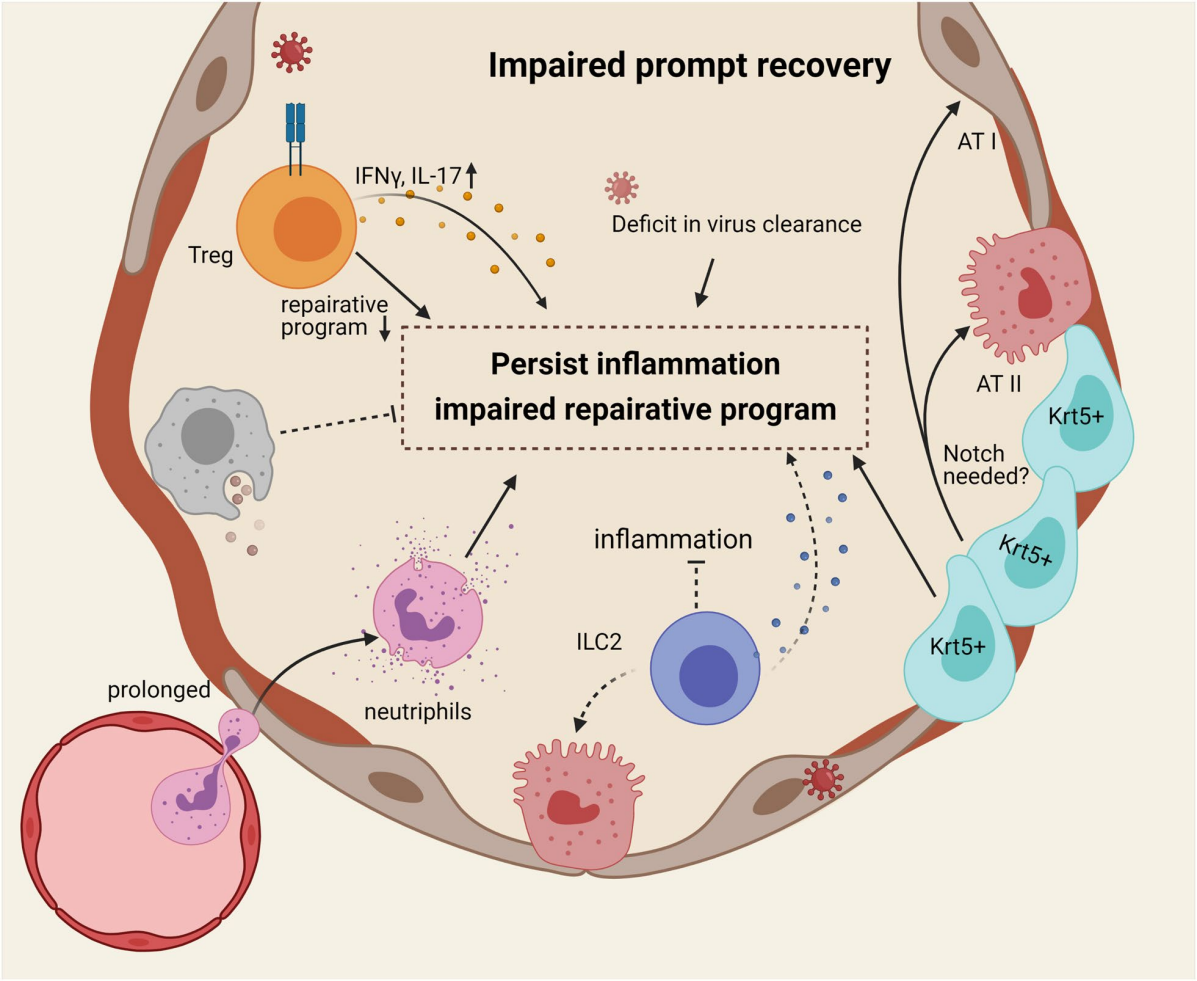
Aged hosts display persistent inflammation and prolonged acute recovery following respiratory virus. The course of the respiratory infection in the aged host is characterized by prolonged recovery. Such impaired recovery program is in part attribute to the retention of neutrophils and the malfunction of AMs’ phagocytosis. The phenotypic skew of Tregs from reparative towards inflammatory is also important in the impaired recovery. Aged lungs also harbor significantly less ILC2s than the young lungs, accompanied by deficit in their pro-repair function. Aside from the immune compartment, ineffective regeneration of epithelial cells in aged hosts also contributes to their impaired reparative program. Krt5 + progenitor cells have been shown to accumulate in the aged lung following influenza virus infection

Besides their defects in mounting effective antiviral immune responses, aged hosts exhibit delayed recovery from acute morbidity compared to the young hosts (Fig. 3). It has been shown that while viral replication may be similar between young and aged lungs, viral clearance is delayed in aged mice compared to the young mice [31]. It is thus possible that the deficit in viral clearance hinders the acute recovery of infected aged hosts. Additionally, prolonged inflammation in aged hosts may hamper such processes. For instance, increased neutrophil levels and prolonged neutrophil infiltration of the lungs were observed in aged hosts. Importantly, neutrophil depletion following influenza infection promotes host survival and lung repair without compromising the viral clearance of the aged hosts [32], suggesting that prolonged neutrophil-mediated damage is a major driver of the impaired recovery from influenza infection in aged hosts.

﻿Aside from the unresolved inflammation, the dysregulated immune cell driven pro-healing program also contributes to the prolonged recovery observed in the aged host. Regulatory T (Treg) cells are important in the pro-resolution and pro-healing process following influenza virus infection [53]. Of note, aging results in a cell-autonomous impairment of reparative Treg cell function during influenza pneumonia. This is at least in part due to the age-related DNA methylation program, leading to the loss of the reparative transcriptional regulatory network of Treg cells during recovery from influenza-induced lung injury in aged hosts. Moreover, these age-associated Treg cells displayed an inflammatory phenotype characterized by the increased expression of Th1 and Th17 transcription factors and signature cytokines IFN-γ and IL-17 [54]. Aside from lacking an antigen-receptor, Innate lymphoid cells (ILC) functionally resemble T cells. Group 2 innate lymphoid cells (ILC2s) are the predominant ILC subset in the lung shown to promote epithelial cell proliferation and tissue repair following influenza-mediated damage [55]. Notably, aged lungs harbor substantially fewer ILC2s and those ILC2s from aged lungs are functionally compromised, failing to produce cytokines at homeostasis and during influenza infection. Importantly, the transfer of activated ILC2s from young mice reduced lung inflammation and damage in aged hosts during influenza infection, suggesting that reviving the ILC2 compartment promotes acute recovery. Additionally, the proper function of lung macrophages is vital for lung inflammation resolution and tissue repair [56–58]. As discussed above, AMs in the aged hosts showed a dysfunctional phenotype in phagocytosis. Such defect would abrogate the clearance of debris and apoptotic cells, which is essential for lung repair [19]. Thus, it is reasonable that malfunctioning AMs during aging may contribute to the delayed healing process displayed in the aged hosts.

Proper regeneration of the stromal cells is not only important for restoration of the air-blood barrier and lung function, but also essential in ceasing the deleterious immune response post virus clearance. It has been shown that total ATII cells and endothelial cells were substantially decreased in the aged hosts. Instead, the accumulation of Krt5 + progenitor cells has been observed following influenza infection [59]. The exact roles of Krt5 + cells in lung repair during influenza virus infection are still controversial. In one report, Krt5 + cells have been shown to accumulate in the lung-damaged areas, and may give rise to both ATI and ATII cells following severe influenza infection. [59] However, another study has demonstrated that those Krt5 + cells failed to give rise to the ATI and ATII cells in the lung unless Notch signaling was inhibited [60]. Furthermore, the accumulation of those Krt5 + cells may represent a “non-functional” repair process of the lungs and contribute to the development of persistent lung pathology such as fibrosis. Currently, little is known regarding the cross-talk of the dysregulated immune function and the development and differentiation of those Krt5 + progenitor cells during aging. However, a better understanding of the cellular and molecular interactions of immune cells and those Krt5 + or Krt5− lung progenitors following lung viral injury may provide insights into the development of future strategies aiming to promote the complete recovery of lung tissue following viral damage during aging.

## Aging and the development of pulmonary sequelae following acute viral infection

**Fig. 4:**
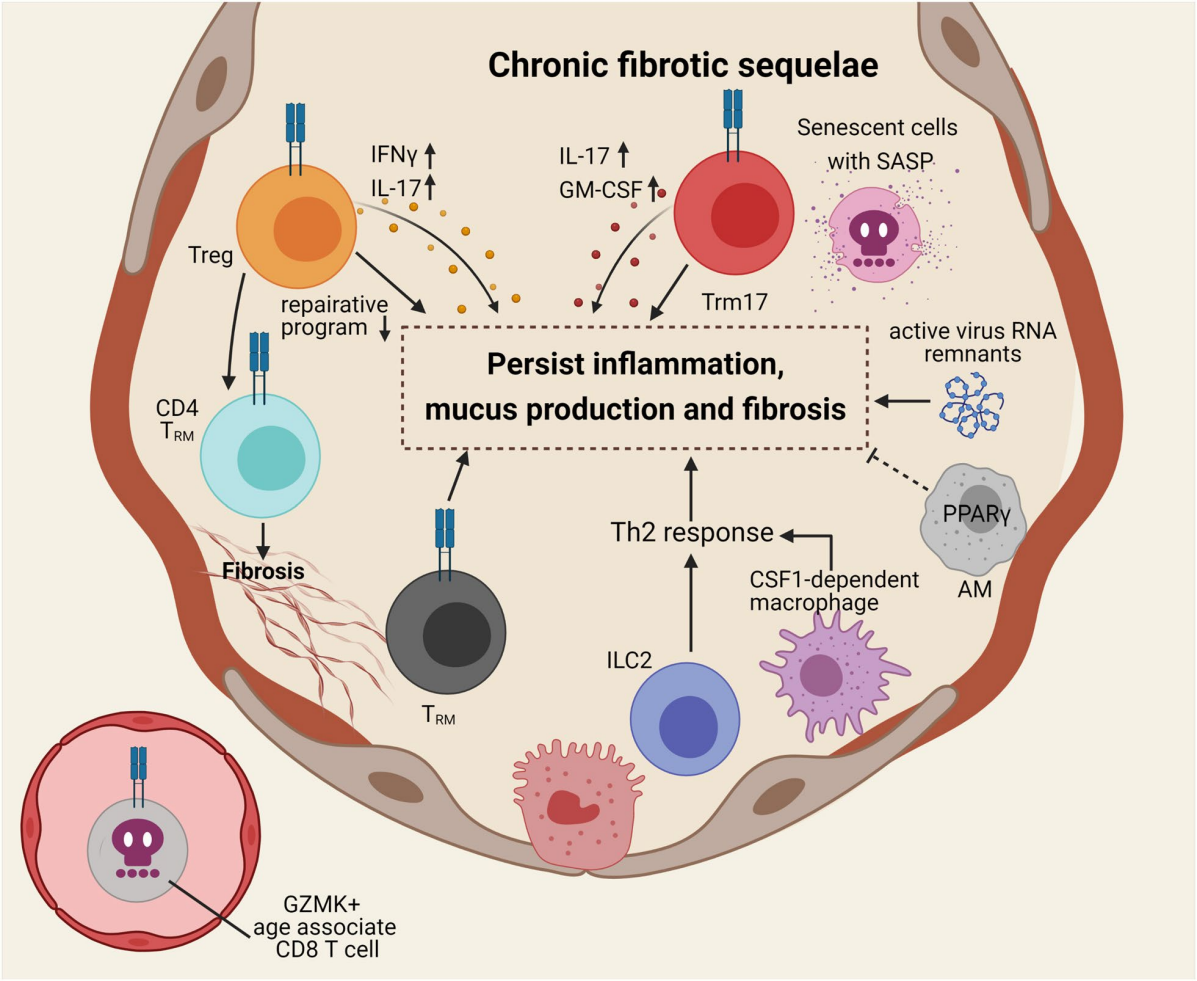
Aging is associated with the development of pulmonary sequelae following respiratory virus clearance. Acute respiratory viral infection can lead to persistence of inflammatory, pathological and pro-fibrotic responses in the aged lung. Active virus RNA remnants could promote non-resolving inflammation. Loss of PPAR-γ function in macrophages could also contribute to the persistent lung fibrotic sequelae. Extensive accumulation of T_RM_ cells in aged lungs fail to provide protective anti-viral response upon secondary challenge. Instead, these T_RM_ cells could induce bystander tissue inflammation and pathology, which drive the development of non-resolving inflammatory and fibrotic sequelae. Dysfunctional T_reg_ could contribute to the loss of regulation in pro-fibrotic responses through CD4-T_RM-_dependent manner. Another CD4 population, which was reported to produce pro-inflammatory cytokines, was found in the BAL of COVID-19 patients and termed Trm17. Trm17 is also considered to interact with pro-fibrotic macrophages and thus contribute the pro-fibrotic response in the lung. Other potential candidates that could be involved in the development of chronic sequelae following acute virus infection include cells that are involved in the TH2 response and cells that undergo cellular senescence

Besides the acute morbidity and mortality induced by respiratory viral infections, recent evidence has revealed that acute respiratory viral infections can lead to persistent inflammatory, pathological and pro-fibrotic responses in the aged lung (Fig. 4). To this end, respiratory viral infection has been identified as a major risk factor for the development and exacerbation of chronic obstructive pulmonary disease (COPD) [61, 62]. A retrospective study has shown that acute viral pneumonia was an independent risk factor for Post-Inflammatory Pulmonary Fibrosis (PIPF) [63]. The follow-up HRCT images of discharged survivors of influenza virus (H7N9) infection showed that although the inflammation was revolved in the first 6-months post discharge, the interstitial fibrosis or ground-glass opacities persisted even after 24 months post-discharge [64]. Additionally, lung fibrosis was well-documented in convalescent of coronavirus infections including SARS-CoV and MERS-CoV infections [65]. Indeed, examination of pathology from lung autopsy samples has revealed extensive evidence of injury and fibrosis that resembled end-stage pulmonary fibrosis in deceased COVID-19 patients [66], suggesting that severe COVID-19 may cause the development of fibrotic lung diseases. Influenza-induced chronic lung sequelae in young mice are mild and self-limited, while severe persistent inflammatory and fibrotic sequelae were reported in the aged hosts [54, 67].

Both the innate and adaptive immune compartments have been shown to contribute to the pathogenesis of the long-term sequelae following respiratory virus infection. Experimentally, respiratory viral infections including influenza virus infection in mice have resulted in persistent inflammation, mucus production and fibrosis weeks after the recovery from acute morbidity [56, 60, 68–70]. The induction of chronic lung diseases following influenza virus infection appeared to be constrained to sites of viral RNA remnants, suggesting that persistent stimulation of innate immune pathways by viral RNA PAMPs may promote chronic disease development following acute viral infection [71]. Intriguingly, the deficiency of PPAR-γ in myeloid cells resulted in persistent lung fibrotic sequelae three months after the primary infection, indicating a potential role of macrophage PPAR-γ function in suppressing such pathogenesis [56].

Among the adaptive immune cells, the CD8 T cells play a vital role in contributing to the long term fibrotic sequalae following respiratory virus infection. Tissue-resident memory (T_RM_) cells are a group of memory T cells that reside in the mucosal tissue. Compare to the circulating memory T cells, T_RM_ cells constitutively express high levels of effector molecules, which confers CD8 T_RM_ cells with superb antiviral function at the cost of potential induction of bystander tissue inflammation and pathology [72]. The exacerbation of chronic lung conditions following acute infection is associated with extensive accumulation of T_RM_ cells in aged lungs, despite a decrease in circulating memory T cell quantity. Interestingly, such accumulation of T_RM_ cells in aged lungs failed to provide substantial protection against influenza rechallenge, suggesting that these T_RM_ cells become dysfunctional during aging. On the contrary, the exuberant T_RM_ accumulation in aged lungs drives the development of non-resolving inflammatory and fibrotic sequelae. High dose of anti-CD8 treatment (depletion of both circulating memory T cells and lung T_RM_ cells), but not a low dose of anti-CD8 treatment (depletion of only circulating memory T cells only but not lung T_RM_ cells) following the recovery of acute infection decreased lung inflammation and collagen deposition [67]. Additionally, the heightened expression of PD-1 by CD8 tissue-resident memory T cells (T_RM_) is also vital for suppressing the chronic lung sequelae following influenza infection. To this end, we found that the persistence of influenza antigen caused high levels of PD-1 expression in a group of influenza-specific T_RM_ cells, which in turn regulate the pathogenicity of these T_RM_ cells in the memory phase [70].

Additionally, post-viral lung fibrosis could involve multiple players aside from CD8 T cells. In this regard, defective Treg function has been linked to the development of age-associated chronic lung conditions following influenza infection [54]. Notably, Treg function has been shown to limit the profibrotic function of CD4 T_RM_ cells in an allergic model [73]. Thus, it is plausible to reason that impaired Treg function during aging may contribute to the exaggerated accumulation of T_RM_ cells in the lung, leading to exuberant development of chronic inflammatory. Moreover, dose-dependent chronic lung disease has been reported in the influenza virus infection model in a Th2 response dependent manner [71]. Consistently, ILC2 and CSF1-dependent macrophages were involved in the asthma-like disease in the chronic phase post SeV infection [74]. Treatment with senolytic drugs, dasatinib plus quercetin (DQ), could rescue lung fibrosis induced by bleomycin injury, suggesting a potential role of senescent cells in the pathogenesis of the chronic sequelae following lung injury during aging. With the flourish of “-Omics”, we are gaining insights into the complex cellular and molecular networks modulating the development of post-viral chronic lung diseases with much better resolution than ever before. It is expected that we will be able to identify druggable targets for preventing and/or treating post-viral chronic lung conditions in the near future.

## Aging and COVID-19

As of May 2021, the pandemic caused by severe acute respiratory syndrome coronavirus 2 (SARS-CoV-2) has caused more than 3 million deaths globally (Johns Hopkins COVID-19 Dashboard “https://coronavirus.jhu.edu”). Aging has been identified as the most prominent risk factor for the development of severe COVID-19 [2, 75]. COVID-19 has a unique pathogenesis pattern where the immune system plays a central role. It was shown that the SARS-CoV-2 virus load was similar in the nasopharyngeal swabs across age, suggesting that the increased susceptibility towards severe diseases upon SARS-CoV-2 infection in the elderly cannot be fully explained by viral replication and spread [76]. Consistently, in the lung tissue of patients who suffered from prolonged severe COVID-19, although the pathology reviewed intensive tissue injury and lung fibrosis, the SARS-CoV-2 virus RNA was not detected, indicating that persistent tissue injury and fibrosis may be mainly caused by dysregulated host responses rather than direct virus-mediated damage [66].

Currently, little is known about how aging influences host immunity against SARS-CoV-2 infection, but unbiased analysis has revealed that aging is generally associated with the enhanced pro-inflammatory profile in the plasma of COVID-19 patients [77]. For instance, GM-CSF, a proinflammatory cytokine that promotes monocyte and dendritic cell responses, is significantly upregulated in aged COVID-19 patients compared to young patients [78]. Consistently, during the incipient stage, an increased proportion of monocytes and DCs, accompanied by lymphopenia, was observed in the PBMC of aged COVID-19 patients when compared to the young [79]. Conversely, CD8 cytotoxic T cell responses, especially in effector memory and terminally differentiated effector CD8 cells, were diminished in COVID-19 patients with advanced age, while CD4 T cell responses were relatively normal [80]. Additionally, although aged COVID-19 patients can mount robust class-switched antibody responses against SARS-CoV2 infection, the effector activity of those antibodies (i.e. FCγ receptor engagement) may be diminished with progressing age [81]. Thus, the aberrant activation of innate inflammation coupled with impaired development of effective adaptive immunity is likely a major cause of the increased acute host morbidity and mortality in severe COVID-19 patients.

Acute respiratory distress syndrome (ARDS) could lead to chronic sequalae even after viral clearance. At 6-month post symptom onset, 76% of COVID-19 patients were reported to have at least 1 persisting symptom [82]. Pulmonary diffusion abnormality and increased percentage change of CT score 6 months post symptom onset were associated with increased disease severity during the acute phase of COVID-19 [82]. There have been limited reports on the likely cellular and molecular mechanism that may drive chronic lung pathologies during aging. Increased expression of immune markers was associated with poor outcome [77], including type 1 immunity markers like IFN-γ in T cells and inflammation-related markers like MP3K8 and IL-1β in monocytes, was observed in the aged “recovered” COVID-19 patient [79]. Although the exact cell type is not specified, circulating T cells in the young “recovered” COVID-19 patients showed increased CD8 T cell “stemness” related markers like TCF7, while the aged had increased senescence markers like CDKN1A as well as GZMK [79], a molecule that promotes SASP release from stromal cells [83]. A resident T cell population producing IL-17 and GM-CSF, termed ﻿tissue-resident memory-like Th17 cells (T_RM_17 cells), have been identified in the BAL from COVID-19 patients 3 weeks post symptom on-set [84] (Fig. 4). It was suggested that T_RM_17 cells interact with the pro-fibrotic macrophages, which could then contribute to long-term sequalae post respiratory viral infection. To this end, certain macrophage populations including those monocyte-derived alveolar macrophages were shown to promote lung fibrosis following bleomycin and influenza-induced lung injury models [85]. Besides CD4 T_RM_ cells, whether CD8 T_RM_ cells could contribute to the development of chronic lung sequelae following COVID-19 during aging, as what have demonstrated in influenza pneumonia model [67], requires further investigation.

One of the barricades for elucidation of the mechanism underlying SARS-CoV2 pathogenesis during aging is the lack of aged animal models for COVID-19. It was reported that the SARS-CoV-2 can actively replicate and cause pathology in the lower airway of non-human primates, ferrets and hamsters [86]. However, the role of progressing age in regulating host susceptibility and disease development was not examined. A promising small animal model of COVID-19 is the human ACE2–transgenic (hACE2-transgenic) mice for SARS-CoV2 infection [87]. However, how aging influence the SARS-CoV-2 infection in such model has not been fully explored. A mouse-adapted SARS-CoV-2 virus strain (SARS-CoV-2 MA10) can replicate in the lower airways of wildtype young and middle-aged (12-month-old) BALB/c mice [88, 89], and causes exacerbated inflammation and lung injury in the later. However, this model has not been adapted to the C57BL/6 genetic background where transgenic and respiratory viral models of aging are more widely studied.

## Conclusion and perspective

Aging leads to increased susceptibility to viral infection, elevated acute morbidity and mortality and increased risk of chronic pulmonary sequelae. Vaccination would still be the primary and best method to prevent infection-induced diseases in the elderly. However, viral mutations may dampen the efficacy of existing vaccines and immune-remodeling in the elderly continues to be a threat for the induction and maintenance of robust vaccine-induced immunity during aging. Therefore, the prompt development of therapeutics for respiratory viral infection is still relevant for unvaccinated elderly populations and for viral infections breaching vaccine-induced protection. Anti-viral drugs, monoclonal antibodies, and steroids are promising in shortening hospitalization time in severe infections. However, the therapeutic effects of these regimens are still limited. Here we have reviewed recent literature in the related areas and discussed the underlying immunological mechanisms underlying the increased susceptibility, acute disease and chronic sequelae following respiratory viral infection during aging. We believe that a better understanding on the cellular and molecular networks regulating the development of age-associated acute morbidity and chronic sequelae following respiratory viral infection is required for advancing novel host-targeting therapeutics that can dampen post-viral acute and chronic conditions during aging.

It is important to note that the altered immune response is only one layer in the complication of viral infection in the elderly. Aging is a risk factor for many comorbidities including cardiovascular disease, diabetes, fibrosis, chronic obstructive pulmonary disease (COPD) and cancer etc [75]. Furthermore, aging is likely accompanied by multiple episodes of prior infectious experiences. Currently, most of our mechanistic understanding about aging and the respiratory viral infection is gained from lab animal models, which are free of aging-related co-morbidities or prior experiences of infection. To better mimic the human immune response, more “natural” models of aging that can mimic age-related comorbidities and/or prior experiences in microbial exposure are needed for establishing more physiologically-relevant animal models. Such studies are expected to gain more precise mechanistic knowledge on the roles of the aging process in modulating host susceptibility to severe acute and chronic disease development in the elderly.

## Data Availability

Not applicable.
